# Infectious Diarrhea in Kidney Transplant Recipients

**DOI:** 10.7759/cureus.82194

**Published:** 2025-04-13

**Authors:** Yassin Loucif, Collin Mackenzie, Olga Tselikmann, Lars C Rump

**Affiliations:** 1 Nephrology, Universitätsklinikum Düsseldorf, Düsseldorf, DEU; 2 Infectious Disease, Universitätsklinikum Düsseldorf, Düsseldorf, DEU

**Keywords:** clostridioides difficile, cytomegalovirus, immunosuppression, infectious diarrhea, kidney transplantation, pathogen spectrum

## Abstract

Introduction

Infectious diarrhea represents a significant and frequent complication among kidney transplant recipients, primarily due to the immunosuppressive therapy required to prevent graft rejection. This condition poses substantial risks to both graft function and patient survival, driven by increased susceptibility to opportunistic pathogens and potential medication-related gastrointestinal effects. This study aims to characterize the pathogen spectrum and associated risk factors of infectious diarrhea in a German cohort of kidney transplant recipients, providing insights into regional patterns and clinical implications.

Methods

A retrospective cohort study was conducted, analyzing 604 patients, including 436 kidney transplant recipients, who were hospitalized with infectious diarrhea (ICD-10 codes A00-A09) at the Universitätsklinikum Düsseldorf (UKD) between January 2019 and December 2023. Nontransplant patients (n = 168) were included as a comparison group to evaluate pathogen distribution and infection risk specific to immunosuppressive therapy in transplant recipients. Pathogen identification was performed focusing on stool samples. Data collected included transplantation status, dates of admission and transplantation, recurrence rates, and detailed immunosuppressive regimens. Statistical analyses were applied to evaluate pathogen distribution, temporal patterns, and the influence of immunosuppression on infection risk.

Results

The most prevalent pathogens identified among kidney transplant recipients were *Clostridioides difficile* (26.3%), cytomegalovirus (CMV, 12.3%), enteropathogenic *Escherichia coli* (EPEC, 8.6%), and norovirus (4.8%). Immunosuppression significantly heightened infection susceptibility, with *Clostridioides difficile* infections occurring notably more frequently and CMV-related diarrhea observed exclusively in the transplant cohort. Recurrence rates were elevated for both CMV and *Clostridioides difficile*, underscoring their clinical persistence. Temporal analysis revealed a median onset of CMV infections at approximately 13 months post-transplantation, with no significant seasonal variation.

Conclusions

The predominance of opportunistic pathogens such as *Clostridioides difficile* and CMV in kidney transplant recipients reflects the profound impact of immunosuppression on infection risk and pathogen profiles. These findings emphasize the necessity for enhanced diagnostic approaches in kidney transplant recipients, including early and comprehensive pathogen screening, and targeted prevention strategies, such as optimized and maybe prolonged CMV prophylaxis and stringent hygiene protocols for *Clostridioides difficile*. It emphasizes close monitoring of a potential viral load in blood samples, especially after cessation of the routine CMV prophylaxis after kidney transplantation. This study contributes to a better understanding of infectious diarrhea in this vulnerable population and lays the groundwork for improved clinical management, overall cost reduction, and future prospective research.

## Introduction

In Germany, more than 2,000 kidney transplants are performed annually, and over 10,000 patients remain on the national transplant waiting list [1]. Kidney transplant recipients are at increased risk for infectious complications due to long-term immunosuppressive therapy, with estimates suggesting that up to 70% of all transplant recipients experience at least one infectious complication within the first three years after transplantation [2]. Gastrointestinal symptoms, particularly diarrhea, are among the most frequent clinical issues in this population. The pathogen spectrum of infectious diarrhea can vary substantially depending on regional epidemiology, immunosuppressive regimens, and individual patient characteristics. Data from India suggest a wide variety of microbial agents, including bacteria, viruses, and parasites [3]. However, there is a significant lack of data on the specific microbial pathogens that lead to hospitalization in kidney transplant recipients within Central Europe.

The aim of this study is to systematically identify and characterize the pathogens associated with both acute and chronic forms of infectious diarrhea in hospitalized kidney transplant recipients. In addition, we assessed patient-specific clinical parameters to determine potential risk factors and relevant clinical associations. A further focus was placed on differentiating infectious from drug-induced toxic diarrhea, which remains a common diagnostic challenge in transplant medicine.

Identifying the most relevant pathogens in this vulnerable patient group may help reduce diagnostic delays between symptom onset and microbiological confirmation. In the long term, this could support the implementation of efficient, targeted, and resource-conscious diagnostic strategies. This study contributes to closing an important research gap and to improving both clinical care and the cost-effectiveness of patient management.

## Materials and methods

Study design

This retrospective cohort study was performed at the Universitätsklinikum Düsseldorf (UKD), Nephrology Department. Patients hospitalized from January 1, 2019, to December 31, 2023, with an infectious diarrhea diagnosis (ICD-10 A00-A09) were included if clinical records were complete. Data were sourced from the GCM Medico hospital information system.

Patient cohort

The cohort comprised 604 hospitalized patients, including 436 kidney transplant recipients (436/604; 72.2%). In addition to kidney transplant recipients, a smaller group of nontransplanted patients with infectious diarrhea was included for comparative purposes of the pathogen spectrum. Collected variables included transplantation status, transplantation date, admission date, identified pathogens, recurrence status, immunosuppression regimens, and demographics (age, gender). Patients with incomplete records were excluded.

Microbiological analysis

Pathogen detection was conducted at UKD’s Institute of Microbiology and Virology. Stool samples underwent DNA extraction with the Qiagen EZ1 DNA Tissue Kit, followed by real-time polymerase chain reaction (PCR) using fluorescence-labeled probes targeting *Clostridioides difficile* toxin genes, cytomegalovirus (CMV) DNA, enteropathogenic* Escherichia coli* (EPEC) intimin gene (eae), and norovirus RNA.

Statistical analysis

Data were processed using Apple Numbers and analyzed with the DATAtab software. Fisher’s exact test was used to compare pathogen frequencies between transplant and nontransplant groups, the Kruskal-Wallis Test was used to assess seasonal clustering, and Mood’s median test was used to evaluate time from transplantation to infection.

Statistical significance was defined as p < 0.05. Numerical values are presented as N, %, or mean ± standard deviation (SD), as indicated. Where applicable, test statistics (e.g., χ²-value, t-value, F-value) are provided alongside the corresponding p-value in tables. Decimal separators were standardized using periods (e.g., 1.25).

## Results

Among 436 transplant recipients, a pathogen was detected in 276 cases. The identified pathogens are presented in Table 1.

**Table 1:** Distribution of detected enteropathogens among hospitalized patients with infectious diarrhea (January 2019-December 2023) EAEC: enteroaggregative *Escherichia coli*;* *EHEC: enterohemorrhagic *Escherichia coli* Absolute (N) and relative (%) frequencies of  enteropathogens detected in stool samples from hospitalized patients with infectious diarrhea

Pathogen	Absolute frequency (N)	Relative frequency (%)
Clostridioides difficile	96	34.78
Cytomegalovirus	74	26.81
Enteropathogenic *Escherichia coli*	40	14.49
Norovirus	28	10.14
Campylobacter jejuni	7	2.54
EAEC	6	2.17
Adenovirus	6	2.17
EHEC	5	1.81
Rotavirus	5	1.81
Astrovirus	3	1.09
Yersinia enteroclitica	2	0.72
Microsporidien	1	0.36
Kryptosporidien	1	0.36
Salmonella spp. B	1	0.36
Salmonella typhimurium	1	0.36
Total	276	100.0

Impact of immunosuppression

Standard immunosuppressive therapy consisted of a combination of calcineurin inhibitors (cyclosporine or tacrolimus) or mTOR inhibitors (sirolimus), mycophenolate mofetil, and corticosteroids (triple therapy). In total, n = 374 patients (85.98%) received triple therapy. Dual immunosuppressive therapy (either a calcineurin inhibitor or mTOR inhibitor combined with corticosteroids) was documented in n = 52 cases (11.95%). Monotherapy with corticosteroids alone was observed in n = 9 patients (2.07%).

Immunosuppressive therapy was significantly associated with an increased risk of infection. Patients receiving immunosuppressive therapy showed a significantly higher incidence of *Clostridioides difficile* infections compared to non-immunosuppressed patients (Fisher’s exact test, test statistic = 0.208, p = 4.08 × 10^-^⁹). CMV was exclusively detected in kidney transplant recipients (test statistic = ∞, p = 1.45 × 10^-^¹¹).

Temporal patterns

**Figure 1:**
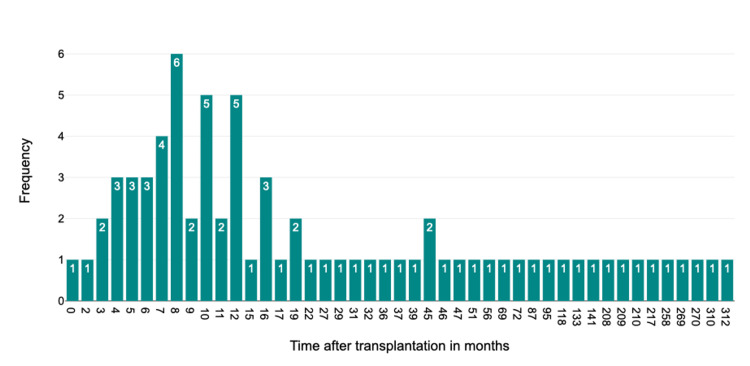
Time-dependent occurrence of cytomegalovirus-associated infectious diarrhea following kidney transplantation CMV: cytomegalovirus Histogram of CMV-positive diarrhea cases (N = 74) by months after kidney transplantation. A statistically significant clustering was observed at 13 months post-transplantation (Mood’s median test, test statistic = 9.63, p = 0.0019)

CMV infections occurred at a median of 13.5 months post-transplantation, coinciding with the end of standard antiviral prophylaxis. This was significantly earlier than the median for all other pathogens (Mood’s median test, test statistic = 9.63, p = 0.0019). *Clostridioides difficile *infections also occurred significantly earlier, with a median of 15.5 months post-transplantation (Mood’s median test, test statistic = 6.54, p = 0.0106). Other pathogens showed no significant temporal clustering (Figure 1).

Seasonal variation

No statistically significant seasonal variation in pathogen detection was observed across calendar months (Kruskal-Wallis test, test statistic = 11.00, p = 0.443). This indicates a uniform distribution of infectious diarrhea cases throughout the year, with no evidence of temporal clustering.

## Discussion

Key findings

This single-center study conducted at the University Hospital of Düsseldorf identified *Clostridioides difficile*, CMV, EPEC, and norovirus as the predominant pathogens causing infectious diarrhea in kidney transplant recipients. Immunosuppressive therapy significantly increased both infection and recurrence risk, with CMV being exclusively detected among transplant recipients (Fisher’s exact test, test statistic = ∞, p < 0.001). The absence of seasonal variation (Kruskal-Wallis test, test statistic = 11.00, p = 0.443) suggests that immunosuppression may be a more relevant risk factor than environmental exposure.

Literature context

These findings are consistent with prior studies reporting EPEC and norovirus as frequent pathogens in kidney transplant recipients [4] and confirming *Clostridioides difficile* as a major cause of infectious diarrhea in this population [5]. Interestingly, the peak incidence of CMV infections at approximately 13 months post-transplant contrasts with earlier studies describing peaks within 30 to 90 days post-transplantation [6]. Parasitic infections, commonly reported in other studies [7], were rarely detected in this cohort, potentially due to regional differences, improved hygiene standards, or the specific diagnostic PCR panel used.

Clinical implications

Multiplex PCR proved effective in identifying causative pathogens and is recommended as a standard diagnostic tool in immunocompromised patients [4]. Enhanced screening for CMV and *Clostridioides difficile* is particularly warranted following the cessation of prophylaxis, especially within the first two years post-transplantation. Implementing rigorous antibiotic stewardship and infection control protocols may help reduce the recurrence of *Clostridioides difficile*-associated diarrhea [8]. Given that persistent diarrhea is associated with an increased risk of graft dysfunction, careful adjustment of immunosuppressive regimens, particularly involving agents such as mycophenolate mofetil, should be considered [9].

Limitations

As a retrospective single-center study, this analysis is subject to several limitations. Selection and information bias cannot be fully excluded. Patients without sufficient clinical or microbiological documentation, such as missing discharge summaries or absent pathogen testing, were not included in the final analysis; however, the exact number of excluded cases was not recorded. There is no specific International Classification of Diseases (ICD) coding for all diarrheal episodes, and microbiological testing was not performed systematically in all cases, but rather based on clinical judgment at the time of presentation. Therefore, it is not possible to determine how many patients may have experienced diarrhea without undergoing microbiological testing. Data on prior antibiotic use, duration of diarrhea, recent travel history, and temporal changes in immunosuppressive therapy were not extracted and could not be analyzed due to inconsistent or incomplete documentation in the medical records. Likewise, we did not evaluate comorbidities such as cardiovascular or metabolic conditions, as they were not part of the study’s predefined focus. Medications known to predispose to diarrhea or *Clostridioides difficile *infection, such as proton pump inhibitors or mycophenolate mofetil, were not systematically assessed. These aspects may warrant further investigation in future prospective studies with standardized data collection.

Future directions

Prospective studies are needed to better assess the impact of extended antiviral prophylaxis and to explore the utility of microbiota-based therapeutic approaches [10]. Multicenter trials may help identify regional pathogen patterns and validate the findings of this study across diverse transplant populations.

## Conclusions

Infectious diarrhea remains a significant complication among kidney transplant recipients, primarily due to the effects of immunosuppressive therapy. This study identified *Clostridioides difficile*, CMV, EPEC, and norovirus as the most frequently detected pathogens. The findings emphasize the importance of targeted diagnostic approaches and the timely identification of high-risk patients. Advanced diagnostic tools such as multiplex PCR, alongside individualized preventive and therapeutic strategies, are essential to preserve graft function and improve long-term patient outcomes.
